# Eating Patterns, Chronotypes, and Their Relationship with Metabolic Health in the Early Postpartum Period in Women after Gestational Diabetes Mellitus

**DOI:** 10.3390/nu16111588

**Published:** 2024-05-23

**Authors:** Anna Lesniara-Stachon, Mariana Treviño Montemayor, Tinh-Hai Collet, Magali Andrey, Dan Yedu Quansah, Jardena J. Puder

**Affiliations:** 1 Obstetric Service, Department Woman-Mother-Child, Lausanne University Hospital, University of Lausanne, 1011 Lausanne, Switzerland; anna.lesniara-stachon@chuv.ch (A.L.-S.); mariana.trevino-montemayor@chuv.ch (M.T.M.); magali.andrey@chuv.ch (M.A.); dan.quansah@chuv.ch (D.Y.Q.); 2 Service of Endocrinology, Diabetes, Nutrition and Therapeutic Education, Department of Medicine, Geneva University Hospitals (HUG), 1211 Geneva, Switzerland; tinh-hai.collet@hug.ch; 3 Diabetes Centre, Faculty of Medicine, University of Geneva, 1211 Geneva, Switzerland

**Keywords:** eating patterns, chronotype, metabolic health, postpartum, gestational diabetes mellitus

## Abstract

Observational studies have shown a relationship between eating patterns and chronotypes with metabolic health in the general population and in healthy pregnancies. Data are lacking in the postpartum period, which is characterized by an externally driven misalignment of sleep and food intake. We investigated the associations between eating patterns, chronotypes, and metabolic health in the early postpartum period in women who had gestational diabetes mellitus (GDM). We prospectively included 313 women who completed their 6–8 weeks postpartum visit between January 2021 and March 2023 at the Lausanne University Hospital. Women filled questionnaires on the timing of food intake, sleep (a shortened Pittsburgh Sleep Quality Questionnaire), and the chronotype (the Morningness–Eveningness Questionnaire) and underwent HbA1c and fasting plasma glucose measurements. After adjustments for weight, sleep quality, or breastfeeding, the later timing of the first and last food intake were associated with higher fasting plasma glucose and HbA1c levels 6–8 weeks postpartum (all *p* ≤ 0.046). A higher number of breakfasts per week and longer eating durations were associated with lower fasting plasma glucose levels (all *p* ≤ 0.028). The chronotype was not associated with metabolic health outcomes. Eating patterns, but not the chronotype, were associated with worsened metabolic health in the early postpartum period in women with previous GDM.

## 1. Introduction

Eating patterns are often used to describe one’s individual eating timing, eating duration, and frequency within a day (such as the number of eating occasions per day or whether breakfasts are consumed) [1]. The relationships and interactions between nutrition, biological rhythms, and metabolic health are generally coined in the umbrella term “chrononutrition” [2], which has gained interest in recent years. Individuals with an evening chronotype typically have later activities, later bedtimes, and later food intake when compared to those with a morning chronotype [3,4]. Growing evidence outside of pregnancy demonstrates a relationship between eating patterns [5,6,7,8,9,10], chronotypes [11], and metabolic health. These studies suggest that shifting one’s food intake towards late evening, eating over a prolonged period per day, skipping breakfast, a lower eating frequency, and an evening chronotype are associated with unfavorable health outcomes [5,9,10,11]. Specifically, late evening food consumption is associated with an increased risk of glucose intolerance [5]. Similarly, breakfast skipping and a lower number of eating occasions per day are related to a higher prevalence of obesity [9]. Furthermore, an evening chronotype is associated with an increased risk of obesity, diabetes, metabolic syndrome, and adverse cardiovascular health outcomes [3,11,12,13].

During pregnancy, physiological changes in maternal metabolism affect fat storage in early pregnancy, followed by increased insulin resistance, maternal glucose levels, and free fatty acids in late pregnancy [14]. They are also influenced by individual eating patterns (eating timing, eating duration, eating frequency like the number of breakfasts per week or the number of eating occasions per day), and by the individual chronotype [15]. Prospective studies on the relationships between eating patterns and metabolic health during pregnancy revealed that increased nocturnal energy intake in the third trimester is associated with increased gestational weight gain (GWG) [16], while longer nocturnal fasting intervals and a lower number of eating occasions per day are related to decreased levels of fasting glucose and 2 h post-OGTT glucose in the second trimester [17]. Pregnant women who have a higher nocturnal energy intake are more likely to skip breakfast and exhibit poorer glucose control, i.e., increased HbA1c, insulin resistance, and insulin level [18]. Pregnant women with an evening chronotype tend to consume breakfast later and have a higher energy intake in the evening compared to those with a morning chronotype [19]. They also have a higher GWG in early [20] or late pregnancy [19].

A higher GWG is associated with an increased risk of pregnancy complications such as gestational diabetes mellitus (GDM) [21]. GDM is a state of glucose intolerance with the first onset during pregnancy that does not meet the criteria of overt diabetes [22]. Women with GDM face a 7–10-fold higher risk of developing diabetes after pregnancy [23] and are more prone to future cardiovascular disease [24]. Notably, within the GDM population, those with an evening chronotype exhibit poorer sleep quality and more depressive symptoms, along with an elevated risk of obstetric complications such as preeclampsia [25]. Furthermore, the chronotype may influence their metabolic health, as evidenced by a study implementing chrononutrition and sleep hygiene interventions among women with GDM, resulting in improved glycemic control [26]. These findings highlight the impact of eating patterns and chronotype on metabolic health in the general population, as well as during pregnancy. This is particularly relevant among metabolically high-risk populations of women with GDM.

In addition to eating patterns and the chronotype, sleep disturbances, such as poor sleep quality and abnormal sleep duration, influence metabolic health including glucose control [27,28]. Insufficient sleep and circadian misalignment, which is defined as “wakefulness and food intake occurring when the internal circadian system is promoting sleep” [29], affect metabolic health including higher glucose, insulin, and triglyceride levels, and are associated with increased risks of diabetes, weight gain, and obesity [30,31,32,33]. In pregnancy, misalignments of eating time with day–night cycles are associated with higher postpartum weight retention (PPWR) [34]. Circadian misalignment is especially prevalent in the postpartum period when caring for the newborn impacts on the parents’ sleep and eating habits. The postpartum period is also characterized by a lower total sleep time and regularity, reduced sleep efficiency, and more frequent awakenings [35,36], all of which influence maternal health after delivery. On the other hand, breastfeeding has been shown to help with sleep regulation for both the mother and child [37], and thus may be considered a protective factor.

Yet, studies in the postpartum have only investigated the role of the chronotype on sleep and mood [38,39]. However, we are not aware of any study examining the relationship between eating patterns and chronotypes on metabolic health in the postpartum period. As women with a history of GDM are at higher risk for developing diabetes, investigating these associations in this population is particularly relevant. In this study, we aimed to investigate the effects of (1) eating patterns and of (2) the chronotype on metabolic health in the early postpartum among women with previous GDM. We also evaluated if these relationships are independent of sleep quantity or quality, or other factors such as breastfeeding or weight.

## 2. Materials and Methods

### 2.1. Study Design and Patient Population

This study is part of an ongoing longitudinal cohort of women with GDM that started in 2011. We invited pregnant women diagnosed with GDM according to the International Association of the Diabetes and Pregnancy Study Groups [22,40], who were attending the antenatal diabetes care at the Woman-Mother-Child department at the Lausanne University Hospital (CHUV) to participate. All women signed an informed consent prior to participation. The study protocol was approved by the Ethics Committee of the Canton de Vaud (326/15) on 17 September 2015.

### 2.2. Inclusion and Exclusion Criteria

For this analysis, we included women with GDM aged ≥ 18 years, who were followed up at our Woman-Mother-Child department between January 2021 and March 2023, and who completed the Timing of the Food Intake (TFI) Questionnaire, the Pittsburgh Sleep Quality Index (PSQI), and the Morningness–Eveningness Questionnaires (MEQ) 6–8 weeks postpartum [41,42].

**Figure 1:**
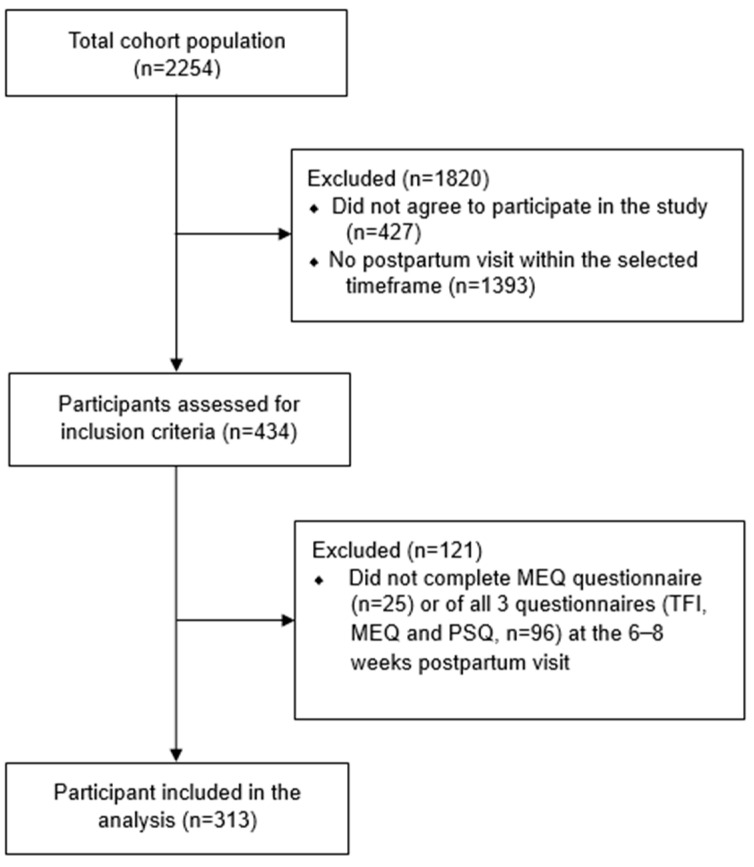
The flow chart of the study participants. TFI: Timing of the Food Intake Questionnaire, MEQ: Morningness–Eveningness Questionnaire, PSQI: Pittsburgh Sleep Quality Index.

As the above-mentioned questionnaires were introduced in January 2021, we excluded out of the total cohort population of 2254 women with GDM those who did not sign an informed consent (*n* = 427) and those who did not or were yet to attend their 6–8 weeks postpartum visit between January 2021 and March 2023 (*n* = 1393). Of the remaining 434 patients, we excluded those who did not complete all three questionnaires (TFI, MEQ, and PSQI questionnaires) at the 6–8 weeks postpartum visit. Thus, the final sample included 313 women (see Figure 1).

## 3. Measurements

### 3.1. Sociodemographic and Medical Characteristics

Data on maternal socio-demographic characteristics, including age, ethnic origin, and educational level, were collected during the first GDM visit that took place after diagnosis if GDM was diagnosed at 24–32 weeks of gestation [43]. Information on the family history of diabetes (first or second degree), previous history of GDM, smoking status during pregnancy, alcohol consumption, gravida, parity, glucose-lowering medical treatment during pregnancy, and breastfeeding status (yes/no) at 6–8 weeks postpartum were extracted from the participants’ medical charts.

#### 3.1.1. Predictors

##### Assessment of the Eating Patterns

We employed a seven-item Timing of Food Intake (TFI) Questionnaire, developed by our team (Appendix A.1), to assess eating patterns, including (a) eating timing, (b) duration, and (c) frequency at 6–8 weeks postpartum. The assessment of the eating timing included the time of the first and the last food intake, the time of the last main meal, and the time of the first and the last drink intake. The questionnaire also assessed the eating frequency with the number of eating occasions per day and the number of breakfasts per week. Finally, we assessed the time of the first and last calorie intake (covering both food or calorie-containing drinks), and we calculated the eating duration as the time interval between the first and last food intake.

##### Assessment of the Chronotype

We evaluated the chronotype of participants using the Morningness–Eveningness Questionnaire (MEQ) at 6–8 weeks postpartum. The MEQ is a self-rated 19-item questionnaire on human circadian rhythms, and categorizes people into morning, evening, and intermediate chronotypes [41]. The MEQ total score ranges from 16 to 86, with scores ≤ 41 considered as eveningness, scores between 42–58 as intermediate/neutral chronotype, and scores ≥ 59 as morningness. The analyses of the MEQ were conducted with the chronotype categories (cutoff values above) and with the continuous value.

##### Assessment of Sleep

The PSQI [42] measures sleep quality over the past month and consists of seven components as follows: subjective sleep quality, sleep latency, sleep duration, sleep efficiency, sleep disturbances, use of sleep medication, and daytime dysfunction. The global score ranges from 0 to 21, with higher scores indicating poorer sleep quality. In this study, we used seven out of the nineteen individual questions, and calculated three out of the seven component scores, i.e., subjective sleep quality (component 1), sleep duration (component 3), and sleep efficiency (component 4) at 6–8 weeks postpartum. Each component score ranges from 0 (no difficulty) to 3 (severe difficulty). In addition to the analysis of the PSQI component, we examined the rise time (in hours), bedtime (in hours), sleep duration (in hours), and sleep efficiency (number of hours slept divided by the number of hours in bed, presented as a percentage, ranging from 0 to 100%).

#### 3.1.2. Outcomes Measures

##### Anthropometric Data

We measured the height and weight of participants during the visit at 6–8 weeks postpartum. Weight and BMI before pregnancy were taken from participants’ medical charts or, if rarely missing, were self-reported. Height and weight were measured to the nearest 0.1 cm and 0.1 kg, respectively, using regularly calibrated electronic scales (Seca^®^ Model 7017021094, Hamburg, Germany)). The BMI was calculated as the weight in kilograms divided by the square of height in meters (kg/m^2^).

##### Metabolic Health Variables

At 6–8 weeks postpartum, we measured the HbA1c level (%) in the venous blood using a high-performance liquid chromatography method (HPLC) according to international guidelines [44]. We also measured fasting glucose, both to reassess glucose control after GDM.

### 3.2. Data Analysis

All analyses were conducted using Stata 15.0 (StataCorp LLC., College Station, TX, USA). Descriptive variables were described as means (±SD) or percentages (%) where appropriate (Table 1, Appendix A Table A1). Notably, all outcome variables (BMI, fasting glucose, HbA1c level) exhibited a normal distribution.

For our first aim, we employed linear regression analyses to investigate cross-sectional associations between the independent variables of interest (eating patterns: timing of the first and last food/calorie intake, time of the last main meal intake, eating duration, number of breakfasts per week, and number of food intakes per day) and the metabolic health outcome variables (BMI, fasting glucose and HbA1c level at 6–8 weeks postpartum, see Table 2). In the first model, we did not adjust for any potential confounders. If relationships were significant in the first model, we performed a second model, where we adjusted for sociodemographic and medical characteristic variables in cases where they were significantly correlated with metabolic health outcomes at 6–8 weeks postpartum. These potential confounding variables included age, breastfeeding, sleep quality (PSQI component 1), sleep duration (in hours and PSQI component 3), sleep efficiency (in % and PSQI component 4), rise time (h) and bedtime (h), and, for fasting glucose and HbA1c as metabolic health outcomes, weight.

For the second aim, linear regression analyses were employed to investigate the relationship between the MEQ score and metabolic health outcome variables at 6–8 weeks postpartum (see Table 3). The first model was unadjusted. If the first model was significant, the same variables (listed above) were tested as potential confounders. If they were significantly related to the outcome variables, they were included in Model 2 (Table 3). We also tested if the MEQ score was associated with eating patterns, specifically the timing of the first and last food/calorie intake, the timing of the last main meal, the eating duration, the number of breakfasts per week, and the number of food intakes per day at 6–8 weeks postpartum. Again, we used an unadjusted model, Model 1, and an adjusted model, Model 2, as mentioned above (see Table 3). To assess circadian misalignment, we also performed correlations between the MEQ score and the actual rise time and bedtime.

In a supplementary posthoc analysis, we grouped the evening (*n* = 9) and the intermediate/neutral (*n* = 140) into a “non-morning” chronotype to better delineate the differences in circadian rhythms among women with previous GDM. This combined group was thus compared with the “morning” chronotype (*n* = 123) in terms of eating patterns, metabolic health, and sleep (see Appendix A Table A2). All statistical significances were two-sided and were with *p* < 0.05.

## 4. Results

### 4.1. Characteristics of Study Participants

Participants had a mean age of 33.6 ± SD 4.6 years. The pre-pregnancy weight and BMI were 71.3 kg ± 16.4 and 26.4 kg/m^2^ ± 5.7, respectively. Half of the women had a university education, and 32% were of Swiss nationality. The majority had a family history of type 2 diabetes (58.7%) and 24% had a history of previous GDM. Additionally, the majority (64%) of women received insulin treatment during pregnancy. At 6–8 weeks postpartum, over 85% were breastfeeding (Table 1). Characteristics of women categorized as a morning or a non-morning chronotype can be found in Appendix A (Table A1). Briefly, women with a morning and those with a non-morning chronotype did not differ regarding ethnic origins, smoking, alcohol intake, or family or medical history regarding metabolic health.

**Table 1:** Socio-demographic characteristics of study participants.

Variable	All Women
Age (years)	33.6 ± 4.6
**Educational level**	
Obligatory education uncompleted	6 (3.9%)
Obligatory education completed	25 (16.5%)
Upper secondary school diploma	16 (10.5%)
General and professional formation	29 (19.1%)
Higher formation (HES, university)	76 (50.00%)
**Ethnic origin**	
Switzerland	78 (32.0%)
Western Europe	50 (20.5%)
Eastern Europe	34 (13.9%)
Africa	34 (13.9%)
Asia	32 (13.1%)
Latin America	13 (5.3%)
North of America	3 (1.2%)
**Family history of Diabetes Mellitus**	
1st degree	80 (35.6%)
2nd degree	52 (23.1%)
No	93 (41.3%)
**History of GDM ^1^**	
Yes	38 (24.4%)
No	118 (75.6%)
**Smoking status during pregnancy**	
Yes	22 (9.4%)
No	209 (88.9%)
Stopped since knowledge of pregnancy	4 (1.7%)
**Alcohol consumption**	
Occasionally	17 (7.4%)
No	213 (92.6%)
**Gravida**	
1	81 (33.2%)
2	68 (27.9%)
≥3	95 (38.9%)
**Parity**	
0	111 (45.5%)
1	79 (32.4%)
≥2	54 (22.1%)
**Glucose-lowering medical treatment during pregnancy**	
None	76 (36.0%)
Metformin	1 (0.5%)
Insulin	133 (63.0%)
Insulin and metformin	1 (0.5%)
Weight before pregnancy (kg)	71.3 ± 16.4
BMI before pregnancy (kg/m^2^)	26.4 ± 5.7
**Breastfeeding at 6–8 weeks postpartum**	
No	31 (14.8%)
Yes	178 (85.2%)

^1^ GDM—gestational diabetes mellitus.

### 4.2. Relationship between Eating Patterns, the Chronotype, and Metabolic Health in the Early Postpartum

Table 2 describes the relationship between eating patterns and metabolic health in the early postpartum period. In the unadjusted results, a later timing of the first food and calorie intake were associated with higher morning fasting glucose levels (all *p* ≤ 0.010). Furthermore, a later timing of the last food intake was associated with higher HbA1c levels (*p* = 0.046), whereas a longer eating duration and a higher number of breakfasts per week were associated with lower fasting glucose levels (all *p* ≤ 0.028).

We then adjusted for confounders including weight, sleep quality, and breastfeeding at 6–8 weeks postpartum, as they were linked to fasting glucose levels at 6–8 weeks postpartum (all *p* ≤ 0.024). After adjusting for confounders that were significant in the univariate analysis, all relationships remained significant (all *p* ≤ 0.03). We found no significant relationships between eating timing, eating duration, the number of breakfasts per week, or the number of food intakes per day and BMI at 6–8 weeks postpartum (see Table 2).

**Table 2:** Relationship between eating patterns and metabolic health in the early postpartum.

	**BMI (kg/m^2^)** **(β [95% CI])**	**HbA1c (%)** **(β [95% CI])**	**Fasting Glucose (mmol/L)** **(β [95% CI])-Model 1**	**Fasting Glucose (mmol/L)** **(β [95% CI])-Model 2**
Time of the first food intake (h)	0.031 [−0.340, 0.402]	−0.002 [−0.032, 0.028]	0.050 [0.012, 0.087] *	0.050 [0.005, 0.095] * ^1,2,3^
Time of the first calorie intake (h)	0.152 [−0.282, 0.586]	−0.003 [−0.038, 0.032]	0.076 [0.032, 0.119] *	0.0513 [0.007, 0.096] * ^1,2,3^
Time of the last main meal (h)	−0.039 [−0.461, 0.383]	0.006 [−0.029, 0.040]	0.001 [−0.042, 0.045]	N/A
Time of the last food intake (h)	0.022 [−0.345, 0.390]	0.030 [0.001, 0.060] *	−0.007 [−0.045, 0.031]	N/A
Time of the last calorie intake (h)	0.089 [−0.271, 0.449]	0.026 [−0.003, 0.054]	0.017 [−0.020, 0.055]	N/A
Eating duration (h)	−0.001 [−0.270, 0.267]	0.016 [−0.005, 0.038]	−0.031 [−0.058, −0.003] *	−0.014 [−0.049, 0.021] ^1,2,3^
Number of breakfasts per week	−0.300 [−0.620, 0.019]	−0.011 [−0.037, 0.015]	−0.041 [−0.074, −0.008] *	−0.030 [−0.067, 0.008] ^1,2,3^
Number of food intakes per day	−0.032 [−0.425, 0.361]	0.021 [−0.010, 0.052]	0.0003 [−0.040, 0.041]	N/A

* *p*-value < 0.05. Adjusted for ^1^ weight, ^2^ sleep quality, and ^3^ breastfeeding at 6–8 weeks postpartum, as they were related to the respective outcome and the unadjusted model was significant.

The relationships between the chronotype (MEQ score) and metabolic health are shown in Table 3. The mean MEQ score was 57.0 ± 8.3, spanning from neutral/intermediate to morning chronotypes. The MEQ score was not associated with BMI, fasting glucose, or HbA1c at 6–8 weeks postpartum.

**Table 3:** Relationship between the MEQ total score and metabolic health or eating patterns.

	Model 1			Model 2		
Variable	Coef.	95% Conf. Interval	*p*-Value	Coef.	95% Conf. Interval	*p*-Value
Metabolic health						
BMI (kg/m^2^)	0.016	−0.071, 0.103	0.721	N/A		
HbA1c (%)	0.003	−0.004, 0.010	0.366	N/A		
Fasting glucose (mmol/L)	−0.004	−0.013, 0.005	0.392	N/A		
Eating patterns						
Time of the first food intake (h)	−0.076	−0.101, −0.051	<0.001	−0.052 ^1,2^	−0.079, −0.025 ^1,2^	<0.001 ^1,2^
Time of the first calorie intake (h)	−0.059	−0.081, −0.038	<0.001	−0.032 ^1,2^	−0.055, −0.009 ^1,2^	0.007 ^1,2^
Time of the last main meal (h)	−0.034	−0.053, −0.014	0.001	−0.023 ^2^	−0.044, −0.002 ^2^	0.032 ^2^
Time of the last food intake (h)	−0.042	−0.065, −0.019	<0.001	−0.032 ^2,3^	−0.057, −0.008 ^2,3^	0.010 ^2,3^
Time of the last calorie intake (h)	−0.039	−0.063, −0.015	0.001	−0.014 ^2^	−0.036, 0.008 ^2^	0.212 ^2^
Eating duration (h)	0.034	0.00004, 0.067	0.050	N/A		
Number of breakfasts per week	0.061	0.031, 0.091	<0.001	0.054 ^2,4^	0.017, 0.090 ^2,4^	0.004 ^2,4^
Number of food intakes per day	0.021	−0.005, 0.047	0.108	N/A		

Model 1—unadjusted. Model 2—adjusted for ^1^ rise time, ^2^ bedtime ^3^ sleep duration, or ^4^ breastfeeding at 6–8 weeks postpartum, when they were related to the respective outcome and the unadjusted model was significant. N/A denotes not applicable.

### 4.3. Morningness–Eveningness Questionnaire (MEQ) Total Score and Eating Patterns

An earlier chronotype (indicated by a higher MEQ score) was associated with an earlier timing of the first food and the first calorie intake, as well as an earlier time of the last main meal, the last food intake, and the last calorie intake (all *p* ≤ 0.001). Furthermore, a higher MEQ score was associated with an increased number of breakfasts per week (*p* < 0.001). Regarding potential confounders, the rise time was related to the time of the first food and first calorie intake, while an earlier bedtime was linked to an earlier time of the first and last calorie intake, time of the first and last food intake, time of the last main meal intake, and a higher number of breakfasts per week. Additionally, a longer sleep duration was related to an earlier time of the last food intake and breastfeeding to a higher number of breakfasts per week. Adjusting for these confounders did not alter the results, except for the relationship between the chronotype and time of the last calorie intake was no longer significant. However, there were no significant relationships between the chronotype and the eating duration or number of food intakes per day (see Table 3). Regarding the extent of external circadian misalignment, there were low correlations (correlation coefficients 0.29–0.35) between the MEQ total score and the actual rise time or bedtime at 6–8 weeks postpartum.

### 4.4. Supplementary Analysis: “Morning” or ”Non-Morning” Chronotype and Metabolic Health

In our population, only few women (*n* = 9) were considered as having an “evening chronotype”, while the majority had “intermediate/neutral” (*n* = 140) or “morning” chronotypes (*n* = 123). In a supplementary analysis of categorical chronotype (Appendix A Table A2), we regrouped the 149 women as “non-morning” and 123 women as “morning” chronotypes, whose mean MEQ total score were 51.0 ± 5.9 and 64.3 ± 3.8, respectively. Women with a morning chronotype exhibited an earlier time of the first food and the first calorie intake, as well as the last main meal, the last food intake, and the last calorie intake when compared to women with a non-morning chronotype (all *p* ≤ 0.025). Additionally, morning women had a higher number of breakfasts per week (*p* = 0.002), but the number of food intakes was not significantly different between the chronotypes. Regarding metabolic health, there were no significant differences in BMI, fasting glucose, and HbA1c levels at 6–8 weeks postpartum between women with a morning vs. a non-morning chronotype. Regarding sleep-related variables, non-morning women had a later rise time and bedtime, a worse sleep quality, and a lower sleep duration and efficiency when compared to morning women (all *p* ≤ 0.032).

## 5. Discussion

The early postpartum is a period where the mother’s circadian misalignment is affected by the needs and caring for the newborn, thus impacting on both sleeping and eating schedules. In this cohort of women with GDM, eating patterns, but not the chronotype, were associated with some metabolic health outcomes at 6–8 weeks postpartum. Specifically, a later timing of both the first and the last food intake, as well as a later timing of the first calorie intake, were associated with higher fasting glucose or HbA1c levels, indicating a worse glucose regulation. These associations remained significant after adjusting for weight, sleep quality, or breastfeeding. In addition, a higher number of breakfasts per week and a longer eating duration were associated with a lower fasting glucose. We did not observe a significant relationship between the chronotype and metabolic health outcomes.

We found positive correlations between a later timing of the first food intake, the first calorie intake, and the last food intake and a higher fasting glucose and/or HbA1c at 6–8 weeks postpartum. These results remained significant after adjustments for weight, sleep quality, or breastfeeding status. Other potential confounders such as age, rise time, bedtime, sleep duration, and sleep efficiency were not related to metabolic health outcomes in the postpartum period. In addition, a lower number of breakfasts per week and shorter eating durations correlated with higher fasting glucose values. However, these results did not remain significant after adjustments. The number of food intakes per day was not related to metabolic health outcomes. Overall, the timing of food intake and eating patterns had a more pronounced impact on metabolic health than the rise time, bedtime, or sleep duration.

Our results are in part consistent with a study of the general population [5] which showed that a later time of the last food intake was associated with higher HbA1c levels and an increased risk of prediabetes or diabetes, particularly in women. A prospective study of 103,312 participants [8] revealed that a later time of the first food intake was associated with a higher incidence of type 2 diabetes. Among individuals with type 2 diabetes, a later time of the last food intake is prevalent [6] and is linked to higher HbA1c levels [45]. Other studies show that eating patterns such as a shorter eating duration, higher frequency of breakfasts, and higher number of food intakes are related to improved metabolic health [9,46,47]. A recent review [46] showed that time-restricted eating can lead to improvements in glucose control and to weight reduction among people with overweight and obesity, but not among individuals with normal weight. Breakfast skipping is associated with an increased risk of obesity [9] and type 2 diabetes [47]. Indeed, breakfast skipping has been linked to a higher percentage of daily caloric intake later in individuals with type 2 diabetes [48]. Another study showed that less than four eating episodes per day were associated with a higher risk of obesity [9]. Studies in pregnancy relating eating patterns to metabolic health have found an association between a higher night eating syndrome score and higher fasting insulin, HbA1c, and high-density lipoprotein cholesterol [18]. Increased night-fasting intervals and reduced eating episodes per day in pregnancy are associated with lower fasting glucose levels [17]. Collectively, these results regarding eating timing and breakfast frequency are consistent with our study that extends existing data to the postpartum period, where misalignments are particularly present. In our population, there was a low correlation between the chronotype (assessed with MEQ score) and the actual rise time or bedtime. Importantly, the MEQ score explained only around 10% of the actual sleep timing. These findings are consistent with an external misalignment in this postpartum period. The overall findings of the previous study and our study suggest a relationship between a later eating timing and breakfast skipping with adverse metabolic health, especially in the female population. However, data regarding the relationship of eating duration with metabolic health found in previous studies are in contrast to our findings.

Food consumption that is not aligned with natural circadian rhythms have negative effects on cardiometabolic health [31]. An intervention study [49] revealed that a 5 h delayed meal timing influences molecular clocks in peripheral tissues, such as white adipose tissue, and contributes to fluctuations in plasma glucose levels. One possible explanation of higher fasting glucose and HbA1c levels among women with later eating timing might be explained by the higher desynchronization of circadian rhythms in peripheral tissues involved in regulating glucose levels, such as the liver, pancreas, muscle, and white adipose tissue [50]. Other potential mechanisms that could explain the association between later eating timing and poorer glucose control might be a shorter time interval between the eating time and the fasting glucose, the reduction in resting-energy expenditure, fasting carbohydrate oxidation, and decreased glucose tolerance when eating food later, as indicated by a randomized controlled trial [51]. In the postpartum period, similarly as during pregnancy [19], skipping breakfast might potentially lead to increased energy intake later during the day. Conversely, in our study, the lower glucose levels when the eating duration is longer can be attributed to the fact that daily calorie distribution tends to be more balanced when consuming food over an extended period. In analogy with previous data [46], this might be more pronounced in normal weight subjects, and therefore our observed relationship did not remain significant when adjusting for weight.

We did not find a significant relationship between the MEQ total score and metabolic health outcomes among women with previous GDM. This is in contrast to studies performed in other populations or contexts that suggest a link between chronotype and metabolic health [5,20]. Indeed, a recent meta-analysis showed that the prevalence of diabetes type 2 is higher in the evening chronotype than the morning chronotype, and that individuals with an evening chronotype have higher fasting glucose, BMI, and total cholesterol levels in comparison with those with a morning chronotype [52]. Possible reasons for the lack of association in our study may include the small population (*n* = 9) of women with an evening chronotype. Some studies [53,54] found a greater prevalence of morning chronotypes in women compared with men. In addition, having children was found to be the strongest determinant of morning chronotypes among women [54]. This may explain the low prevalence of the evening chronotype in our population. Furthermore, in the postpartum period, when misalignment is particularly pronounced, as the needs of the newborn impact both on the sleeping and eating schedules, the actual eating patterns could have a more pronounced influence on women’s metabolic health than the “theoretical” chronotype preference.

To our knowledge, this is the first study to investigate the relationship between eating patterns, the chronotype, and metabolic health in the postpartum period, which is an important and unique period in women’s life with externally driven circadian misalignment. We investigated a metabolically high-risk population of women with a history of GDM, and we took several relevant confounding factors into account. Our cohort is a clinical multiethnic population. Despite these strengths, our study has some limitations. For example, there was a limited number of participants with an evening chronotype, which may have affected some of our results. In addition, we used questionnaires and not objective measures of sleep or food intake, which were not possible to include in a clinical cohort. The lack of data in our cohort regarding women’s dietary habits, food intake, and physical activity levels represents a further limitation of our study, as we had to limit the number of questionnaires in this clinical population. However, we did not find significant differences in socio-demographic or health characteristics, including ethnic origin, health behavior, a family history of diabetes (first or second degree), a previous history of GDM, or glucose-lowering medical treatment during pregnancy between the morning and non-morning chronotypes. Despite these limitations, our findings highlight the importance of assessing eating patterns in the postpartum period in the management of women after GDM, as they are modifiable risk factors for glucose control management in this population.

## 6. Conclusions

In this prospective cohort of women with GDM, we identified relationships between eating patterns and glycemic control in the early postpartum. Specifically, a later time of food and calorie intake, a shorter eating duration, and a lower number of breakfasts per week were associated with poorer glycemic control, as shown by higher fasting glucose and HbA1c levels. The impact of eating patterns on metabolic health was more pronounced than the one of sleep timing. In a time period where externally driven circadian misalignment is particularly pronounced, we did not find any associations between the chronotype preference and metabolic health. These findings emphasize the importance of including eating patterns as a potential factor in glycemic control strategies in women with a history of GDM. Future studies could enlarge the scope to also include dietary habits, physical activity, and more in-depth evaluations of socio-economic factors as potential confounders or mediators, and could investigate physiological mechanisms such as energy expenditure or fasting carbohydrate oxidation and their impact on metabolic health in the postpartum. There is also a need for intervention trials studying the impact of advancing the timing of food intake and regular breakfast consumption on glucose control in the postpartum period among women with GDM.

## Data Availability

The raw data supporting the conclusions of this article will be made available by the authors on request. The data are not publicly available because it is a clinical data maintained and kept in a secure server at the Lausanne University Hospital.

## Appendix A

**Table A1:** Socio-demographic characteristics of women characterized as morning or as non-morning chronotypes.

Variable	Non-Morning Chronotype (N = 149)	Morning Chronotype (N = 123)	*p*-Value
Age (years)	33.6 ± 4.5	33.8 ± 4.8	0.764
**Ethnic origin**			0.927
Switzerland	41 (34.8%)	33 (35.1%)	
Western Europe	26 (22.0%)	19 (20.2%)	
Eastern Europe	16 (13.6%)	11 (11.7%)	
Africa	13 (11.0%)	13 (13.8%)	
Asia	14 (11.9%)	12 (12.8%)	
Latin America	5 (4.2%)	6 (6.4%)	
North of America	3 (2.5%)	0 (0%)	
**Family history of Diabetes Mellitus**			0.199
1st degree	42 (37.5%)	24 (28.9%)	
2nd degree	23 (20.5%)	20 (24.1%)	
No	47 (42.0%)	39 (47.0%)	
**History of GDM ^1^**			0.756
Yes	17 (25.4%)	13 (21.0%)	
No	50 (74.6%)	49 (79.0%)	
**Smoking status during pregnancy**			0.121
Yes	12 (10.4%)	4 (4.5%)	
No	102 (88.7%)	81 (92.0%)	
Stopped since knowledge of pregnancy	1 (0.9%)	3 (3.5%)	
**Alcohol consumption**			0.282
Occasionally	8 (7.0%)	7 (8.1%)	
No	106 (93.0%)	79 (91.9%)	
**Gravida**			0.503
1	47 (39.8%)	29 (30.9%)	
2	32 (27.1%)	27 (28.7%)	
≥3	39 (33.1%)	38 (40.4%)	
**Parity**			0.386
0	64 (54.2%)	40 (42.6%)	
1	35 (29.7%)	35 (37.2%)	
≥2	19 (16.1%)	19 (20.2%)	
**Glucose-lowering medical treatment during pregnancy**			0.980
None	38 (36.9%)	31 (39.2%)	
Metformin	1 (1.0%)	0 (0%)	
Insulin	63 (61.1%)	48 (60.8%)	
Insulin and metformin	1 (1.0%)	0 (0%)	

^1^ GDM—gestational diabetes mellitus.

**Table A2:** Comparison of metabolic health, eating patterns, and sleep-related variables between women characterized as morning or as non-morning chronotypes.

Variable	Non-Morning Chronotype (N = 149)	Morning Chronotype (N = 123)	Mean Difference	*p*-Value
Metabolic health and MEQ score				
MEQ total score	51.0 ± 5.9	64.3 ± 3.8	−13.2 ± 8.3	<0.001
BMI (kg/m^2^)	27.6 ± 5.5	27.1 ± 5.0	0.5 ± 5.3	0.50
HbA1c (%)	5.3 ± 0.4	5.3 ± 0.4	0.02 ± 0.4	0.70
Fasting glucose (mmol/L)	5.1 ± 0.6	4.9 ± 0.4	0.1 ± 0.5	0.10
Eating patterns				
Time of the first food intake (h)	9.4 ± 1.9	8.6 ± 1.7	0.9 ± 1.8	<0.001
Time of the first calorie intake (h)	8.9 ± 1.4	8.2 ± 1.7	0.7 ± 1.6	<0.001
Time of the last main meal (h)	20.0 ± 1.3	19.7 ± 1.5	0.4 ± 1.4	0.025
Time of the last food intake (h)	21.3 ± 1.7	20.8 ± 1.5	0.5 ± 1.6	0.019
Time of the last calorie intake (h)	21.5 ± 1.7	21.1 ± 1.6	−0.5 ± 1.7	0.020
Eating duration (h)	11.9 ± 2.4	12.3 ± 2.2	−0.4 ± 2.3	0.17
Number of breakfasts per week	5.2 ± 2.4	6.0 ± 1.7	−0.8 ± 2.1	0.002
Number of food intake per day	4.0 ± 1.7	4.2 ± 1.9	−0.2 ± 1.8	0.46
Sleep				
Rise time (h)	7.5 ± 1.5	6.8 ± 1.4	0.7 ± 1.5	<0.001
Bedtime (h)	23.1 ± 1.3	22.5 ± 1.2	0.6 ± 1.3	<0.001
Sleep quality (subscale 1) *	1.6 ± 0.8	1.3 ± 0.9	0.4 ± 0.9	<0.001
Sleep duration (h)	5.7 ± 1.4	6.1 ± 1.5	−0.4 ± 1.5	0.032
Sleep duration (subscale 3) *	1.8 ± 0.9	1.6 ± 0.9	0.2 ± 0.9	0.06
Sleep efficiency (%)	68.2 ± 16.8	72.8 ± 16.0	−4.6 ± 16.5	0.025
Sleep efficiency (subscale 4) *	1.9 ± 1.1	1.6 ± 1.2	0.3 ± 1.2	0.053

* Higher scores indicate a worse sleep quality. Hours (h) are expressed as decimal fractions.

### Appendix A.1. Timing of Food intake (TFI) Questionnaire

During the past week:

(1) At what time is your first meal or first food intake?

TIME ___________

(2) At what time is your last main meal (for example supper)?

TIME ___________

(3) At what time is your last food intake (can be your main meal or any food intake such as snack or fruit, etc.)?

TIME ___________

(4) At what time is your first drink (only for fruit juice, milk, cocoa or sweet drink)?

TIME ___________

(5) At what time is your last drink (only for fruit juice, milk, cocoa or sweet drink)?

TIME ___________

(6) On average, how many food intakes do you have in a day including drinks such as fruit juice, milk, cocoa, sugary drink?

NUMBER OF INTAKES ___________

(7) On average, how many times a week do you eat breakfast?

NUMBER OF TIMES ___________
